# Presepsin Levels in Infection-Free Subjects with Diabetes Mellitus: An Exploratory Study

**DOI:** 10.3390/biomedicines12091960

**Published:** 2024-08-29

**Authors:** Dimitrios Kouroupis, Ioanna Zografou, Aikaterini Balaska, Andromachi Reklou, Anna Varouktsi, Anastasia Paschala, Athina Pyrpasopoulou, Konstantinos Stavropoulos, Konstantinos Vogiatzis, Anastasia Sarvani, Panagiotis Doukelis, Dimos Karangelis, Georgios Dimakopoulos, Kalliopi Kotsa, Michael Doumas, Theocharis Koufakis

**Affiliations:** 1 Second Propedeutic Department of Internal Medicine, Hippokration General Hospital, Aristotle University of Thessaloniki, 546 42 Thessaloniki, Greece; dimcour841@gmail.com (D.K.); ioannazo@yahoo.gr (I.Z.); aikatbal@yahoo.gr (A.B.); machirkl28@yahoo.com (A.R.); annavarouktsi@gmail.com (A.V.); a.pyrpasopoulou@doctors.org.uk (A.P.); konvstavropoulos@hotmail.com (K.S.); vogiatzikos@gmail.com (K.V.); natasasar91@gmail.com (A.S.); pitdukel@yahoo.gr (P.D.); michalisdoumas@yahoo.co.uk (M.D.); 2 Department of Internal Medicine, G. Papanikolaou General Hospital, 570 10 Thessaloniki, Greece; npaschala@yahoo.gr; 3 Department of Cardiothoracic Surgery, Democritus University of Thrace, University General Hospital, 681 00 Alexandroupolis, Greece; dimoskaragel@yahoo.gr; 4 BIOSTATS, Epirus Science and Technology Park Campus of the University of Ioannina, 451 10 Ioannina, Greece; info@biostats.gr; 5 Division of Endocrinology and Metabolism and Diabetes Centre, First Department of Internal Medicine, Medical School, Aristotle University of Thessaloniki, AHEPA University Hospital, 546 36 Thessaloniki, Greece; kalmanthou@yahoo.gr

**Keywords:** diabetes mellitus, presepsin, inflammation, glycemic control

## Abstract

Systemic inflammation has been recognized as the cause and consequence of metabolic dysregulation in diabetes mellitus (DM). Presepsin has recently emerged as a promising biomarker for the detection of bacterial infections and sepsis. There is evidence that gut dysbiosis results in the increased circulating concentrations of Gram-negative bacteria lipopolysaccharide, the linkage of presepsin, which in turn promotes insulin resistance and correlates with the risk of diabetic complications. Thus, we hypothesized that presepsin could reflect the magnitude of systemic inflammation and metabolic decompensation in patients with DM even in the absence of infection. In this cross-sectional pilot study, we included 75 infection-free individuals with well-controlled (*n* = 19) and uncontrolled (*n* = 23) type 2 diabetes (T2D), well-controlled (*n* = 10) and uncontrolled (*n* = 10) type 1 diabetes (T1D), and normoglycemic controls (*n* = 13). Presepsin levels were compared between the groups and potential associations with demographic, clinical, and laboratory parameters were explored. We observed that the duration of DM was associated with presepsin values (*p* = 0.008). When the participants were classified into the type of DM groups, the presepsin levels were found to be lower in the patients with T2D compared to those with T1D (*p* = 0.008). However, significance in that case was driven by the difference between the well-controlled groups. After adjusting for the effects of DM duration, presepsin was significantly lower in the well-controlled T2D group compared to the well-controlled T1D group [1.34 (2.02) vs. 2.22 (4.20) ng/mL, *p* = 0.01]. Furthermore, we adjusted our findings for various confounders, including age, body mass index, and waist circumference, and found that the difference in the presepsin values between the adequately controlled groups remained significant (*p* = 0.048). In conclusion, our findings suggest that presepsin could potentially serve as a surrogate marker of inflammation and metabolic control in people with DM.

## 1. Introduction

Diabetes mellitus (DM) is a pathophysiologically heterogeneous group of chronic metabolic disorders characterized by elevated plasma glucose levels and associated with life-threatening complications and high morbidity and mortality rates [1]. In type 2 diabetes (T2D), which is the most common form of the disease, insulin resistance, relative insulin deficiency, impaired incretin effect, upregulation of glucagon levels, and increased renal glucose reabsorption are key pathophysiological features. On the contrary, in type 1 diabetes (T1D), the T-cell-mediated autoimmune destruction of pancreatic beta cells leads to complete insulin deficiency. Regardless of the differences in the pathogenesis of the two main types of diabetes, a growing amount of evidence suggests that systemic inflammation is an interface between T1D and T2D, being the cause or consequence of metabolic dysregulation in both cases [2]. More specifically, a bidirectional link has been reported between hyperglycemia, inflammation, and diabetic complications, mediated by complex pathways that involve oxidative stress, the accumulation of advanced glycation end products, and endothelial dysfunction, among others [3].

Presepsin has recently emerged as a promising prognostic marker for bacterial infections, sepsis, and septic shock [4]. CD14, expressed on the surface of monocyte/macrophage, belongs to the Toll-like receptor (TLR) family and is involved in the recognition of pathogen ligands, including the Gram-negative bacteria lipopolysaccharide (LPS) [5]. CD14 contributes to the presentation of LPS to TLR, resulting in a cascade of intracellular signals that activate the expression of the genes responsible for the immune response. The soluble form of CD14 has been named presepsin and its levels are considered a marker of activated innate immune effector cells in response to invasive pathogens [6]. Although presepsin is normally detected in noninfective individuals, its concentrations increase in the early stages of bacterial infections and correlate with the intensity of innate immune activation [7]. Compared to other well-established inflammation biomarkers, such as C-reactive protein (CRP) or procalcitonin (PCT), presepsin is believed to be a better detector of bacterial infection due to its direct involvement in relevant pathogenetic pathways [8].

Several studies have investigated the role of presepsin in the prediction of infection and sepsis among DM patients and have shown good diagnostic accuracy [9,10]. However, data on presepsin levels are lacking in people with DM who are free from infection. It is well established that DM affects the gut microbiota balance by increasing systemic inflammation. This is the case, for example, with inflammatory bowel diseases (IBDs), which have an inflammatory and immune-mediated pathogenetic background, where DM exacerbates the inflammatory state and disease progression. In a recent meta-analysis, Fuschillo et al. [11] showed that DM could negatively affect the clinical course of IBD by increasing the risk of hospitalization and infections. Tanimura et al. [12] demonstrated elevated plasma presepsin concentrations in infection-free patients with systemic lupus erythematosus (SLE) compared to controls, indicating the inappropriate activation of monocytes and neutrophils in the pathophysiology of SLE exacerbation. Taken together, these data suggest a complex pathophysiological interplay between metabolic, inflammatory, and autoimmune disorders, of which many aspects remain vague. Interestingly, evidence from animal and human studies indicates that intestinal dysbiosis observed in DM results in elevated circulating LPS concentrations by altering the epithelial barrier and increasing intestinal permeability, a phenomenon known as metabolic endotoxemia [13]. The existing data suggest that LPS levels increase in uncontrolled diabetes and correlate with the risk of diabetic complications [14,15], setting the theoretical background for a link between DM and presepsin even in the absence of infection.

In this context, we hypothesized that presepsin levels could reflect the magnitude of systemic inflammation and metabolic dysregulation in people with DM. The main objective of the present pilot study was to explore possible differences in circulating presepsin levels between patients with controlled and uncontrolled T1D and T2D and normoglycemic controls, all without infection. The secondary objective was to identify the associations between presepsin levels and various demographic, clinical, and laboratory parameters.

## 2. Material and Methods

### 2.1. Study Design

This cross-sectional pilot study was carried out in the Second Propedeutic Department of Internal Medicine of Aristotle University in Thessaloniki, Greece, between January and May 2024. The study was carried out according to the principles of the Declaration of Helsinki and its subsequent amendments. All the study participants gave their informed written consent before enrolment in the study, and the study protocol was approved by the Scientific Council of the Hippokration General Hospital of Thessaloniki (approval number 47699/23-10-2023).

### 2.2. Inclusion and Exclusion Criteria

The study inclusion criteria were the following: (i) having received a diagnosis of T1D or T2D from a physician according to the criteria of the American Diabetes Association (ADA) [16], (ii) age over 18 years, and iii. the complete medical history of medications, smoking status, and diabetic complications at the time of inclusion in the study.

Exclusion criteria were as follows: (i) Estimated glomerular filtration rate (eGFR) < 60 mL/min/1.73 m^2^. The reason is that presepsin is excreted from the kidneys; therefore, in patients with renal failure, its concentrations are expected to be higher [17]. (ii) Age over 70 years. This criterion was introduced in light of the previous studies showing that presepsin concentrations increase in people over 70 years of age even in the absence of acute infection compared to younger individuals [18]. (iii) Acute or recent (within the last 3 months) infections, surgical operations, or other severe inflammatory disorders (e.g., pancreatitis, burns, etc.). (iv) A history of diabetic ketoacidosis or/and hyperosmolar hyperglycemic state within the last 3 months. (v) Treatment with agents that can affect body weight and blood glucose levels other than antidiabetics, statins, and antihypertensives (e.g., corticosteroids, antipsychotics, etc.). (vi) Severe liver disease. (vii) A history of autoimmune diseases and/or malignancies. (viii) The presence of diabetes complications (both microvascular and macrovascular) given their strong link to systemic inflammation [19].

The control group consisted of hospital employees who had no history of DM or prediabetes and who were also free of acute or chronic infections and other inflammatory conditions according to the criteria mentioned above. Based on their glycated hemoglobin (HbA1c) values, the participants with DM were classified into well-controlled (<7%) and uncontrolled (≥7%) diabetes groups.

### 2.3. Data Collection

Demographic, anthropometric, and laboratory parameters were recorded for each participant, all on the same day. More specifically, the demographic parameters recorded included sex, age, duration of DM, type of antidiabetic medications, use of statins and antihypertensives, and smoking status (current, past, or never). The anthropometric parameters included body weight, height, and waist circumference (WC). Height was measured with a Holtain wall stadiometer. WC was measured midway between the lowest rib and the iliac crest using an anthropometric tape. Body weight was recorded to the nearest 0.1 kg using a calibrated computerized digital balance (K-Tron P1-SR, Onrion LLc, Bergenfield, NJ, USA). Each participant was barefoot and lightly dressed during the measurement. Body mass index (BMI) was calculated by dividing a participant’s weight in kilograms by their height in meters squared. The anthropometric evaluation was separately confirmed by a second investigator.

Blood samples were drawn in the morning after a 12 h overnight fast by antecubital venipuncture, and the samples were stored at −20 °C prior to analysis. The laboratory parameters measured included complete blood count, HbA1c, fasting glucose, total cholesterol, triglyceride, low-density lipoprotein cholesterol, high-density lipoprotein cholesterol, CRP, serum creatinine, and spot urinary albumin-to-creatinine values. eGFR was calculated using the CKD-EPI equation, as recommended by the most recent KDIGO guidelines [20]. Presepsin was determined in serum using an ELISA kit (Wuhan HealthCare Biotechnology Co., Wuhan, Hubei, China). All the analyses were performed in the same laboratory.

### 2.4. Statistical Analysis

Scale outcomes were described as median [Interquartile Range (IQR)] and categorical using frequencies and percentages. Fisher’s exact test was used to assess treatment, sex, and smoking differences in the study groups and the analysis of variance to assess differences in age, BMI, WC, DM duration, and other continuous variables. Multiple linear regression was applied to determine the effect of the group and other confounders on presepsin levels. The analysis was carried out using SPSS v.28 and the significance level was set at 0.05 in all the cases.

## 3. Results

### 3.1. Characteristics of the Study Population

**Figure 1:**
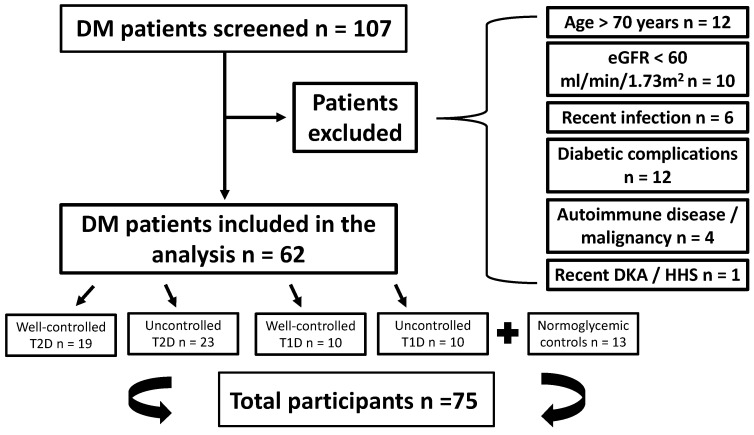
The study selection flow chart. Abbreviations: DM: diabetes mellitus; T1D: type 1 diabetes; T2D: type 2 diabetes; eGFR: estimated glomerular filtration rate; DKA: diabetic ketoacidosis; HHS: hyperosmolar hyperglycemic state.

Seventy-five individuals in total were included in the study divided into five subgroups as follows: well-controlled T2D: *n* = 19; uncontrolled T2D: *n* = 23; well-controlled T1D: *n* = 10; uncontrolled T1D: *n* = 10; and normoglycemic controls: *n* = 13. Figure 1 presents the study selection flow chart.

In terms of demographic characteristics, the patients with well-controlled and uncontrolled T1D were, as expected, younger compared to the participants with well-controlled and uncontrolled T2D [45 (9) and 26 (19) vs. 62.5 (7) and 65 (4) years, respectively; *p* < 0.001]. The patients with well-controlled T2D had a shorter duration of DM compared to the other groups [5 (8.5) vs. 12 (18); 18 (19); 11 (8) years, *p* = 0.037]. On the contrary, there were no significant differences in smoking status and sex distribution.

In terms of anthropometric characteristics, the patients with well-controlled T1D, well-controlled T2D, and uncontrolled T2D had a higher BMI than the participants in the uncontrolled T1D and control groups [26.7 (5.6); 29.9 (6.2); 29.8 (8) vs. 25 (5.5) and 24.7 (3.3) kg/m^2^, respectively; *p* < 0.001]. As expected, the WC values were higher in the patients with poorly and well-controlled T2D compared to the controls and subjects with uncontrolled T1D [106 (13.5) and 105 (8.5) vs. 84 (15) and 89 (14.5) cm, respectively, *p* < 0.001].

The mean HbA1c values did not differ significantly between the patients with poorly controlled T1D and T2D [8.1 (2) vs. 7.7 (0.7) %, *p* = 0.54] and between the well-controlled T1D and T2D groups [6.8 (0.9) vs. 6.1 (0.6) %, *p* = 0.43]. As expected, all the patients in the well-controlled T1D group were treated with insulin. Among the patients with adequately controlled T2D, 84.21% were on metformin therapy, 47.36% on sodium–glucose co-transporter inhibitors (SGLT2i), 31.57% on dipeptidyl peptidase 4 inhibitors, 63.15% on glucagon-like peptide 1 receptor agonists (GLP-1 RA), 5.26% on sulfonylureas (SUs), 5.26% on pioglitazone, and 15.78% on insulin (*p* < 0.001 for all the comparisons versus the T1D group). Reasonably, the use of statins and antihypertensives was more frequent among the participants with adequately controlled T2D compared to those with well-controlled T1D (78.84 vs. 20.00% and 89.47 vs. 10.00%, respectively, *p* < 0.001 in both cases).

Table 1 presents the key characteristics of the study population categorized into study groups.

### 3.2. Presepsin Levels in the Study Groups

In the multiple linear regression analysis, only the duration of DM was significantly associated with the presepsin values (*p* = 0.008) (Table 2).

**Figure 2:**
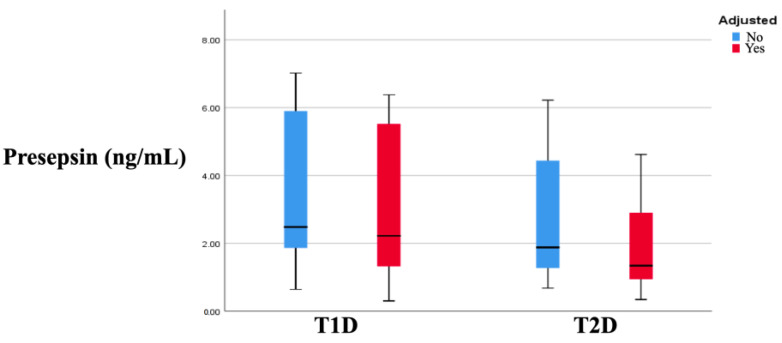
Differences in the presepsin levels between the T1D and T2D groups adjusted and unadjusted for the status of glycemic control. Abbreviations: T1D: type 1 diabetes; T2D: type 2 diabetes.

When the participants with DM were classified into the type of diabetes groups (i.e., T1D and T2D regardless of glycemic control), the presepsin levels were found to be lower in the patients with T2D compared to those with T1D (*p* = 0.008). However, as shown in Figure 2, the significance in that case was driven by the difference between the well-controlled groups.

Given that, as previously mentioned, the duration of DM was associated with presepsin in the regression analysis, we adjusted the difference in the presepsin values between the well-controlled groups for the effects of this parameter and found that the significance remained [1.34 (2.02) vs. 2.22 (4.20) ng/mL, *p* = 0.01]. Furthermore, we adjusted for the parameters that differed significantly between the groups (even though these were not found to be associated with presepsin), including age, BMI, and WC, and found that the difference between the adequately controlled groups remained significant (*p* = 0.048). Table 3 presents the presepsin values in the study groups.

## 4. Discussion

To the best of our knowledge, this is the first study in which circulating presepsin concentrations are measured in infection-free subjects with DM. Given the absence of relevant data in this population, we designed a pilot exploratory study to investigate the factors that influence presepsin levels in order to obtain directions for future, more focused research in the field. We found that presepsin is associated with the duration of DM and is downregulated in patients with well-controlled T2D compared to those with well-controlled T1D.

There is no doubt that DM is a state of low-grade choric inflammation; however, the pursuit of the ideal marker that can effectively reflect the magnitude of inflammation and potentially correlate with the risk of complications is ongoing. The Health, Aging, and Body Composition study demonstrated that dysglycemia is associated with inflammation, as expressed by an increase in CRP, tumor necrosis factor-alpha (TNF-a), and interleukin-6 (IL-6) levels among older people (70–79 years) with prediabetes and DM [21]. Interestingly, there is evidence that elevated CRP levels at birth can predict the later development of T1D among children at high genetic risk, suggesting that T1D is an “immunoinflammatory” disease [22]. Plasma PCT levels, another marker widely used for the diagnosis of bacterial infections, are associated with incident T2D independent of the common factors that predispose to the development of DM, such as adiposity [23]. These findings are in line with our observation regarding the lack of association between presepsin and BMI or WC. On the contrary, the association between CRP and T2D is confounded by central adiposity, the markers of liver dysfunction, and adiponectin levels, indicating that the link between CRP and T2D is largely mediated by ectopic fat accumulation [24].

A systematic review and meta-analysis by Du et al. [25] found a strong correlation between CRP and TNF-a levels with HbA1c and fasting glucose values in patients with T2D. Zhao et al. [26] demonstrated similar findings in Chinese pregnant women with gestational DM, where a positive correlation was established between inflammatory markers and BMI, insulin, and HbA1c. Numerous large epidemiological studies have examined the relationship between CRP and incident T2D, some of them showing a positive association [27,28], while others demonstrated nonsignificant results after adjustment for the markers of adiposity and insulin resistance [29]. The conflicting findings probably reflect the heterogeneity between studies in the included populations, study design, length of follow-up, etc. [30]. Our findings also replicate previous research showing that the degree of inflammation is associated with the duration of DM. In patients with long-standing T2D, Alexandraki et al. [31] observed an upregulation of pro-inflammatory molecules, including IL-6, IL-1β, high-sensitivity CRP, and TNF-a, as well as a higher number of cytokine-secreting cells compared to normoglycemic controls. In individuals with T1D, the duration of DM has been associated with an increase in the number of circulating immune cells and the inflammatory proteome [32].

Salguero et al. [33] have shown that the imbalance in the gut bacteria population in favor of Gram-negative microorganisms in people with T2D and chronic kidney disease (CKD) and the associated increase in LPS levels correlate with inflammatory biomarkers, such as CRP, TNF-α, and IL-6. In support of the notion that the two main types of DM share several common pathophysiological features, Aravindhan et al. [34] showed that chronic endotoxemia (defined as high LPS levels) is seen in subjects with T1D many years before the onset of microvascular complications and is accompanied by increased levels of IL-6, IL-1, and TNF-a compared to normoglycemic controls. A growing body of evidence suggests that metabolic endotoxemia is involved in the progression and development of diabetic retinopathy, one of the leading causes of blindness worldwide, through various pathways, including the exacerbation of vasculopathy and neurodegeneration [35].

Given that many aspects of the pathophysiological links between presepsin and DM are still obscure, it is difficult to provide a definitive explanation of the key finding of the present study, which is the lower presepsin levels in controlled T2D compared to controlled T1D. However, it can be hypothesized that our observation is related to the anti-inflammatory effects of the pharmaceutical agents used in the management of T2D. Metformin, which was used by most of our study population, has been shown to significantly decrease CRP levels [SMD: −0.76 mg/L; 95% CI (−1.48, −0.049); *p* = 0.036] in people with T2D [36]. Similar effects have been demonstrated for SGLT2i [37] and GLP-1 RAs [38] and these are believed to contribute to the impressive cardiorenal protective properties of the newer antidiabetic agents. Even SUs, whose role in T2D therapy is gradually losing ground, have been shown to possess anti-inflammatory properties [39]. Although insulin is also known to exhibit anti-inflammatory actions, these are short-term and can be counteracted in the long term by increasing body weight that fuels the vicious cycle of inflammation [40]. Furthermore, insulin therapy in individuals with T1D has been associated with increased glycemic variability, which in turn aggravates inflammation and oxidative stress [41].

A systematic review by Gomes et al. [42] suggested that antidiabetic medications decrease fasting LPS levels, with the strongest effect induced by rosiglitazone and the weakest by insulin. Statins are also effective in downregulating systemic inflammation, as shown by their potential to reduce CRP levels in patients with established cardiovascular disease [43]. Consistent with our findings, Biyik et al. [44] found that presepsin levels are not elevated in patients with controlled hypertension, another chronic disease related to subclinical inflammation, and the authors attribute this observation to the anti-inflammatory properties of antihypertensive drugs.

The strengths of our study lie in its novelty and the fact that we excluded the participants with CKD, older age, and diabetic complications, which are factors known to interfere with inflammation status and/or presepsin levels. However, its findings should be interpreted in light of important limitations. The cross-sectional design is unable to establish causal associations, while the small number of participants and the exploratory pilot character render its findings only hypothesis-generating. The heterogeneity in patient characteristics between the T1D and T2D groups was unavoidable due to intrinsic differences between the two main types of DM and their therapy. For example, T2D presents at a much older age than T1D and this explains the shorter duration of the disease in the well-controlled T2D group compared to the respective T1D group. However, the presepsin values were not found to be associated with most of the features that differed significantly between the groups, and statistical adjustments for several confounders confirmed the consistency of our observations. Although an analysis investigating the effects of drugs on presepsin levels would be of interest, we are afraid that this was not feasible, as most of the participants with T2D were under a combination of at least two different medications that overlapped between the patients. Combination regimens are a common practice in T2D according to the current guidelines given the progressive nature of the disease. Therefore, it was not feasible to isolate specific drugs that could be associated with presepsin concentrations. Furthermore, the effects of drugs on presepsin levels should ideally be investigated under a different study design, i.e., a prospective study to measure presepsin before and after the treatment administration. We intend to further explore the effects of pharmacotherapy on presepsin concentrations in a larger interventional study, as well as investigate the prospective association between presepsin and diabetic complications.

## 5. Conclusions

In conclusion, our findings suggest that presepsin could potentially serve as a surrogate marker of inflammation and metabolic control in people with DM. It appears to be associated with the duration of the disease and is downregulated in patients with adequately controlled T2D. However, the pathophysiological connections between DM and presepsin, as well as how pharmacotherapy, including newer antidiabetics, antihypertensives, and statins, mediates the above relationship, warrant further investigation in future studies.

## Figures and Tables

**Table 1:** Key characteristics of the study population categorized into study groups.

Group						
Parameter	Controls (*n* = 13)	Uncontrolled T1D (*n* = 10)	Well-Controlled T1D (*n* = 10)	Uncontrolled T2D (*n* = 23)	Well-Controlled T2D (*n* = 19)	*p*-Value for Difference between Groups
Age (years)	55 (8)	26 (19)	45 (9)	65 (4)	62.5 (7)	<0.001
Female sex (%)	46.2	40	10	37.5	40	0.443
Duration of DM (years)	-	11 (8)	18 (19)	12 (18)	5 (8.5)	0.037
Current smokers (%)	7.7	28.6	30	13	26.3	0.755
BMI (kg/m^2^)	24.7 (3.3)	25 (5.5)	26.7 (5.6)	29.8 (8)	29.9 (6.2)	<0.001
WC (cm)	84 (15)	89 (14.5)	103 (17)	106 (13.5)	105 (8.5)	<0.001
HbA1c (%)	5.4 (0.3)	8.15 (2)	6.8 (0.9)	7.7 (0.7)	6.15 (0.6)	0.445
ACR (mg/g)	17 (0)	10.2 (7)	9.1 (11.7)	6 (14.2)	11.5 (16)	0.765
CRP (mg/dL)	0.7 (1.6)	1.65 (2.3)	1.45 (2.4)	1.0 (2)	1.9 (1.2)	0.232
TChol (mg/dL)	230 (76)	162 (34)	158.5 (36)	155 (72)	150.5 (51)	0.040
Metformin (%)	-	0	0	86.95	84.21	<0.001
SGLT2i (%)	-	0	0	39.13	47.36	<0.001
DPP4i (%)	-	0	0	34.72	31.57	<0.001
GLP-1 RAs (%)	-	0	0	43.47	63.15	<0.001
SUs (%)	-	0	0	17.39	5.26	<0.001
Pioglitazone (%)	-	0	0	8.69	5.26	<0.001
Insulin (%)	-	100	100	39.13	15.78	<0.001
Statins (%)	7.69	30	20	82.6	78.84	<0.001
Antihypertensives (%)	15.38	20	10	78.26	89.47	<0.001

Abbreviations: T1D: type 1 diabetes; T2D: type 2 diabetes; BMI: body mass index; WC: waist circumference; HbA1c: glycated hemoglobin; ACR: albumin to creatinine ratio; CRP: C-reactive protein; TChol: total cholesterol; SGLT2i: sodium–glucose co-transporter inhibitors; DPP4i: dipeptidyl peptidase 4 inhibitors; GLP-1RAs: glucagon-like peptide 1 receptor agonists; SUs: sulfonylureas. The values are presented as median (IQR) or percentages.

**Table 2:** Multiple linear regression analysis of the relationship between presepsin as the dependent variable and the various parameters studied.

Parameter	Beta Coefficient	*p*-Value
DM duration	0.122	0.008
Age	0.231	0.478
Sex	−0.356	0.987
Smoking	0.045	0.921
BMI	0.291	0.289
WC	0.345	0.812
HbA1c	0.378	0.752
CRP	0.176	0.148
ACR	−0.098	0.781
Tchol	0.051	0.351

Abbreviations: DM: diabetes mellitus; BMI: body mass index; WC: waist circumference; HbA1c: glycated hemoglobin; ACR: albumin to creatinine ratio; CRP: C-reactive protein; TChol: total cholesterol.

**Table 3:** Presepsin levels in the study groups.

Presepsin (ng/mL)			
Group	*n*	Median	IQR
Control	13	2.46	6.66
Uncontrolled T1D	10	2.48	4.04
Well-controlled T1D	10	2.22 *	4.20
Uncontrolled T2D	23	1.88	3.24
Well-controlled T2D	19	1.34 *	2.02
Total of participants	75	2.00	2.50

Abbreviations: T1D: type 1 diabetes; T2D: type 2 diabetes; IQR: Interquartile Range. * Statistically significant difference between the groups (*p* = 0.01) (adjusted for the effects of the duration of diabetes).

## Data Availability

The data presented in the study are available upon request from the corresponding author. The data are not publicly available due to the privacy restrictions of the Greek National Health System.
